# Association of Supportive Text Messaging With Health Service Utilization Costs Following Psychiatric Admission: Secondary Economic Analysis of a Stepped-Wedge Cluster Randomized Trial

**DOI:** 10.2196/93954

**Published:** 2026-08-21

**Authors:** Vincent Israel Opoku Agyapong, Lola Ibraimova, Reham Shalaby, Belinda Agyapong, Wanying Mao, Ernest Owusu, Hossam Eldin Elgendy, Ejemai Eboreime, Peter H Silverstone, Pierre Chue, Wesley Vuong, Shireen Surood, Frank MacMaster, Andrew J Greenshaw, Arto Ohinmaa

**Affiliations:** 1 Department of Psychiatry, Faculty of Medicine, Dalhousie University, 5909 Veterans' Memorial Lane, 8th Floor, Abbie J. Lane Memorial Building, QEII Health Sciences Centre, Halifax, NS, B3H 2E2, Canada; 2 Department of Psychiatry, Faculty of Medicine and Dentistry, University of Alberta, 4-142A Katz Group Centre for Research, 11315 - 87 Ave NW, Edmonton, AB, T6G 2H5, Canada, 1 1780714431; 3 School of Public Health, University of Alberta, Edmonton, AB, Canada; 4 Clinical Operations Informatics Office, Health Shared Services, Edmonton, AB, Canada; 5 Yaq̓it ʔa·knuqⱡi’it First Nation, Grasmere, BC, Canada

**Keywords:** text messaging, peer support, health care, inpatient, emergency department, physician, cost

## Abstract

**Background:**

Psychiatric hospitalizations are a major driver of mental health–related health care costs, with readmission risk and service utilization being highest in the months following discharge. Scalable, low-cost postdischarge interventions that reduce inpatient utilization are therefore of high policy relevance.

**Objective:**

This secondary exploratory economic study used linked administrative health care utilization data to evaluate the economic impact of supportive text messaging (SMS), alone and in combination with peer support services (PSS), following discharge from acute psychiatric care.

**Methods:**

This secondary exploratory economic evaluation was conducted alongside a pragmatic stepped-wedge cluster randomized trial in Alberta, Canada. Adults discharged from acute psychiatric inpatient care received treatment as usual (TAU), SMS, or SMS with or without PSS. Direct health system costs, including hospital care and physician costs, were assessed using administrative data for 6 months and 12 months before and after the index admission. Difference-in-difference analyses were used to estimate cost changes between groups.

**Results:**

At 6 months post discharge, the period of greatest new costs, participants receiving SMS experienced a significant reduction in hospital care costs compared with TAU (mean difference-in-differences −$7147; 95% CI −$12,388 to −$1906; *P*=.01). Total health care costs were also significantly lower in the SMS group at 6 months (−$8160) relative to TAU. Sensitivity analyses demonstrated that the direction and statistical significance of the primary findings remained stable across alternative intervention costing assumptions and analytic specifications. No significant cost differences were observed at 12 months. The addition of peer support did not result in incremental cost savings at either time point.

**Conclusions:**

SMS was associated with significant reductions in hospital care and total health care utilization costs during the 6-month postdischarge period compared with TAU. These findings support the integration of cost-effective and scalable digital interventions into routine psychiatric discharge pathways to help improve system efficiency in publicly funded health systems.

## Introduction

Mental health disorders impose a substantial and growing economic burden on publicly funded health care systems, driven largely by high rates of inpatient service use [1]. Psychiatric hospitalizations are among the costliest components of mental health care, with recurrent admissions and prolonged lengths of stay accounting for a disproportionate share of total expenditures [2]. As demand for acute psychiatric services continues to rise, health systems face increasing pressure to identify interventions that can reduce avoidable inpatient utilization while remaining scalable and cost-efficient.

The period following discharge from psychiatric hospitalization represents a particularly important window for cost containment. Readmission risk is highest within the first 6 months post discharge, and even small reductions in rehospitalization rates or inpatient length of stay can translate into meaningful cost savings at the population level [3]. Inpatient psychiatric care is resource-intensive, requiring specialized staffing and infrastructure, and repeated admissions signal inefficiencies in transitional care pathways. From a health system perspective, reducing early readmissions and shortening inpatient stays are therefore key targets for improving allocative efficiency without compromising patient outcomes [2].

Although a growing body of literature has examined interventions to improve postdischarge outcomes in psychiatric populations, most studies have focused primarily on clinical or patient-reported outcomes, such as symptom severity, recovery, or quality of life, rather than health care utilization outcomes that directly map onto costs [4-6].

Supportive text messaging (SMS) and peer support service (PSS) interventions have emerged as promising approaches that may address this evidence gap [7,8]. Automated SMS programs deliver brief, standardized messages aimed at promoting engagement, coping, and self-management at minimal marginal cost [3,8]. PSSs leverage lived experience to enhance social support and care continuity, potentially reducing reliance on acute services. Both interventions are comparatively inexpensive to deliver and highly scalable, making them attractive candidates for cost-effective postdischarge support [3,9]. While prior studies have demonstrated improvements in psychosocial outcomes associated with these interventions, their impact on utilization-based outcomes relevant to economic evaluation, such as readmissions, has not been well characterized. Building on earlier effectiveness trials, this study evaluates the impact of SMS alone and in combination with PSS on psychiatric readmissions and inpatient length of stay at 6 and 12 months before and after an index hospitalization. As a follow-up to previously published effectiveness results, we conducted a cost analysis to examine health care costs associated with the interventions, using consistent reporting frameworks, outcomes, and analytic principles to ensure comparability with the original trial findings [3,9]. By focusing on utilization outcomes that serve as key drivers of inpatient costs, this study aims to generate evidence relevant to health system decision-making, informing whether low-cost, scalable postdischarge interventions have the potential to reduce the economic burden associated with psychiatric inpatient care.

## Methods

### Study Design and Data Collection

The study design has been described in the published protocol [10] and in a related paper on health care utilization [3]. In summary, this study is a secondary, utilization-focused economic evaluation conducted alongside a pragmatic stepped-wedge cluster randomized trial, undertaken as a planned follow-up to previously published effectiveness analyses. The trial evaluated SMS alone, SMS with or without PSS, and treatment as usual (TAU) among adults discharged from acute psychiatric inpatient care in Alberta [3]. Specifically, the study assessed the cost implications of 2 postdischarge digital intervention pathways compared with usual postdischarge care alone: (1) a fully automated, web-based SMS program (Text4Support; SMS) delivered alongside usual care; and (2) a pragmatic enhanced-support pathway consisting of SMS with or without the addition of PSS. In this second pathway, all participants received Text4Support, while PSS was provided only to a clinically indicated subgroup identified by the inpatient multidisciplinary team as being at higher risk of psychiatric readmission. Eligibility for PSSs was determined pragmatically by the inpatient multidisciplinary team using routine clinical judgment informed by factors such as recent psychiatric admissions, illness severity, psychosocial vulnerability, and anticipated challenges with community reintegration, rather than through the use of a formalized risk assessment tool.

Outcomes of interest were changes in mean health care costs preindex and postindex admission. The index date was defined as the discharge date from the qualifying psychiatric inpatient admission that triggered trial enrollment. Preindex (baseline) observation periods were defined relative to the admission date of the index hospitalization, capturing health care utilization prior to inpatient admission. Postindex follow-up periods were defined relative to the discharge date, reflecting health care utilization following hospital discharge. A pragmatic stepped-wedge cluster-randomized design with 3 study arms was employed across 10 acute-care sites in Alberta, which served as the units of randomization. Participants received usual postdischarge care alone, SMS plus usual care, or SMS with or without PSS plus usual care. This design is well-suited to evaluating complex, large-scale service interventions and change management initiatives [11]. The 3-arm structure enabled the assessment of whether SMS improved outcomes relative to usual care and whether PSS conferred incremental cost benefits beyond SMS alone. The randomization of 4 cluster units was conducted by an independent statistician, correcting an error of 5 clusters reported in the published protocol [10]. The trial was conducted using an unblinded pragmatic design in which participants, clinical staff, peer support workers, and investigators involved in intervention delivery and data collection were aware of study group allocation. Blinding was not feasible because of the nature of the interventions, particularly the direct delivery of SMSs and the active involvement of peer support workers within clinical care pathways. The adoption of an unblinded pragmatic approach was intended to reflect real-world implementation conditions and enhance the external validity and applicability of the findings within routine psychiatric service settings. Administrative health care utilization and cost data were collected for 1 year preindex and postindex admission using deidentified records.

The stepped-wedge cluster randomization was conducted across 4 psychiatric care cluster units representing the 10 inpatient psychiatric service settings in Edmonton, Calgary, and Grande Prairie, Alberta. The stepped-wedge cluster randomized design was selected because the intervention was implemented pragmatically across psychiatric service settings over time, making simultaneous implementation across all sites operationally challenging. This design also allowed all participating clusters to eventually receive the intervention while reducing contamination between study pathways and supporting evaluation under real-world service delivery conditions. Stepped-wedge designs are particularly well suited for evaluating service delivery and health system interventions expected to do more good than harm and when phased implementation is required for logistical or organizational reasons [12]. To minimize spillover effects, interventions were implemented at the cluster-service level during defined study periods, SMS was delivered individually through a secure digital platform, and PSSs were restricted to participants within the designated intervention pathway.

### Ethical Considerations

Ethical approval was obtained from the University of Alberta Health Research Ethics Board (Pro00111459), with regional operational approval. All participants provided informed consent, and the study adhered to the Declaration of Helsinki.

### Study Participants

Participants were recruited from inpatient psychiatry units among adults diagnosed with a mental illness and deemed ready for discharge. In-person recruitment occurred across 10 acute psychiatric care sites in Edmonton, Calgary, and Grande Prairie, Alberta. Operational managers and clinical staff assisted the research team by identifying patients expected to be discharged within 7 days. Eligible patients received detailed study information, provided written consent, and completed a self-administered questionnaire using a tablet device. Inclusion criteria were age 18 years or older, ownership of a mobile phone, ability to read English-language text messages, and capacity to provide informed consent. Patients who anticipated being out of the province during the 12-month follow-up period were excluded. Recruitment took place over 2 years, from February 2022 to February 2024. Participants were allocated to one of 3 intervention groups through cluster randomization, resulting in a total sample of 1155 participants. Psychiatrists and nursing staff identified individuals at higher risk of readmission who were eligible for PSSs, consistent with pragmatic trial principles that incorporate clinical judgment and team-based risk assessment.

### Text and Peer Support Interventions

The text and peer support interventions have previously been reported in the study protocol [10] and related papers [3]. In summary, the Text4Support program, delivered through the ResilienceNHope platform [13], provided evidence-based daily SMSs to individuals discharged from acute psychiatric care. This low-cost intervention, adapted from the Text4Hope program—which enrolled over 50,000 participants during the COVID-19 pandemic [14]—was designed to reduce the psychological treatment gap and was delivered either alone or in combination with PSS. Prior evaluations of Text4Hope demonstrated that automated, web-based SMS effectively reduces psychological distress and achieves high user satisfaction [15-17].

Messages were initiated the day after enrollment and delivered once daily for 6 months in a unidirectional (no-reply) format. Content was developed by mental health clinicians using cognitive behavioral therapy principles in collaboration with individuals with lived experience. Messages included general supportive themes (eg, hope, affirmation, and self-care), as well as diagnosis-specific content tailored to 6 common psychiatric conditions: mood, anxiety, psychotic, substance use, adjustment, and personality disorders. The first message included contact information for crisis services. Participants assigned to Text4Support had their phone numbers registered on the ResilienceNHope platform to receive tailored messages based on their primary diagnosis.

The PSSs were delivered by trained peer support workers employed by Alberta Health Services who had lived experience of mental illness and recovery, thereby providing in-person or virtual support, advocacy, and linkage to community resources and sharing of recovery experiences. The frequency and duration of contact were determined pragmatically within routine service delivery, and peer support workers were selected based on relevant lived experience and recovery expertise to support participant engagement and community reintegration.

### Sample Size Considerations

We estimated that a total sample size of 1051 participants was required to assess the cost-effectiveness of the SMS and PSS interventions on the primary economic outcomes. This estimate assumed a small effect size (0.2) for the reduction in mean health care costs from baseline to study end points, a population variance of 1 for each mean cost estimate, a 2-sided significance level of α=.05, and 90% power (β=.10). The original pragmatic stepped-wedge trial protocol did not include a formal prospective sample size calculation for the economic evaluation; the sample size justification presented in this paper has been included retrospectively to contextualize the statistical power available for the secondary health care cost analyses.

### Outcome Measures

The primary economic outcome was the change in total health care costs per patient from preindex to postindex admission. Secondary outcomes included category-specific costs disaggregated into physician service costs, emergency department (ED) costs, and inpatient hospitalization costs.

Hospital admission costs and ED visit costs were estimated using resource intensity weights (RIWs) applied to standardized cost weights for inpatient care, consistent with the Canadian Institute for Health Information costing methodology [18]. Physician costs reflected reimbursed claim amounts. Costs were aggregated at the patient level for each observation window (6- and 12-mo preindex and postindex admission) and expressed in constant 2025 Canadian dollars, adjusted for inflation. The economic analysis included all randomized participants with complete, linked administrative cost data for the relevant observation windows.

### Economic and Statistical Analysis

The economic analysis adopts a health system perspective, consistent with publicly funded health care delivery in Alberta, Canada, and focuses on direct medical costs borne by the provincial health authority. The analytical framework is based on a difference-in-difference (DID) approach, comparing changes in costs from preindex to postindex hospitalization across intervention groups, and it allows for the estimation of intervention-associated changes in health care expenditures while accounting for baseline differences and secular trends. This approach is grounded in established theoretical and conceptual frameworks of health care utilization and cost of health care utilization associated with adverse psychiatric events. These conceptual frameworks demonstrate that prior adverse events and health care utilization are among the strongest predictors of recurrence of adverse outcomes and subsequent health care use, including self-harm [19], ED visits [20], and inpatient psychiatric admissions [21-24]. For example, patients with a history of psychiatric hospitalization prior to an index admission have been shown to have approximately twice the risk of readmission within 12 months compared with those without such a history [23]. Consequently, this subgroup is also more likely to incur higher acute health care costs in the postindex admission period.

The economic analysis was conducted from a health system perspective, capturing costs associated with physician services, ED visits, and inpatient hospital admissions. Costs incurred outside the health care system (eg, productivity losses, patient out-of-pocket costs, social services) and costs related to privately funded health care services, including sessions with private psychologists and therapists, were not included. In addition, costs associated with interactions with allied mental health professionals within the publicly funded community mental health program were not available for inclusion in this analysis. The time horizon encompassed 6 and 12 months before and after the index psychiatric admission. All cost components were inflated to constant 2025 Canadian dollars using Alberta’s Consumer Price Index adjustment factors [25].

Mental health service utilization data were obtained from administrative health records maintained by the provincial health authority. These data included the following:

- physician claims data, capturing fee-for-service billings for outpatient (community and ambulatory care) and inpatient physician services;
- ED visit records, capturing ED encounters and associated RIW; and
- inpatient hospitalization records, including RIW, were used to estimate hospital costs.

Based on the established theoretical and conceptual frameworks of health care utilization and cost for adverse psychiatric events [19-24], DID estimates were obtained by modeling health care costs as a function of the study group, the time period (pre vs post), and their interaction.

The coefficient on the group-by-period interaction term represents the incremental change in costs attributable to the intervention beyond secular trends observed in the TAU group. ANOVA-based DID models were used as an initial screening tool to identify cost categories exhibiting potential between-group differences. Final pairwise contrasts were then estimated using mean DID values with pooled variance estimates to maintain consistency with the analytic strategy applied in the parent trial and to facilitate the interpretability of absolute cost differences.

### Sensitivity Analysis

To assess the plausibility of the parallel trends assumption underlying the DID analyses, baseline health care utilization costs were compared across study groups during the preindex observation periods. In addition, sensitivity analyses were undertaken to evaluate the robustness of the findings under alternative costing assumptions and analytic specifications. Deterministic sensitivity analyses incorporated fixed per-patient intervention delivery costs into the primary health care cost estimates, including conservative implementation cost estimates (CAD $50 per patient [CAD $1=US $0.71 as of July 20, 2026]) and higher stress-testing assumptions (up to CAD $1000 per patient), to determine whether the observed cost differences remained stable after accounting for intervention costs. Further analyses examined baseline comparability across major health care utilization cost categories and repeated the models using alternative specifications and pooled variance estimators to validate the consistency of the primary findings.

### Missing Data and Cost Attribution

Missing data were handled using a prespecified, rule-based approach consistent with best practices for analyses of Canadian administrative health data [26,27]. No exclusions were applied for high-cost observations, as extreme costs represent a meaningful component of mental health service utilization and are directly relevant for health system budgeting and resource allocation. In this study, health care utilization and cost outcomes were assessed separately across 4 observation periods (12 mo prior to index admission, 6 mo prior to index admission, 6 mo after discharge from index admission, and 12 mo after discharge from index admission). As a result, missingness occurred at the observation-period level rather than at the participant level. Participants were not excluded from the analysis because hospital-related information was unavailable in a given period. Consequently, participants could have valid hospital utilization and cost data in one period and no hospitalization-related data in another period while remaining included in the analysis.

For hospital care costs, a confirmed inpatient admission event was the primary criterion for inclusion in cost analyses. Hospital cost records that did not have a corresponding admission event were excluded, as these observations could not be reliably attributed to an inpatient episode. These exclusions were limited to records lacking an associated admission event and were not based on cost magnitude. Among individuals with a confirmed hospital admission but missing a recorded RIW, hospital costs were imputed using the mean RIW of the corresponding intervention group (TAU, SMS, or SMS with or without PSS) within the same observation period (preindex or postindex). The imputed RIW was multiplied by the provincial cost per weighted case for the relevant fiscal year to derive hospital costs. This group- and time-specific approach preserves valid admission events while maintaining consistency with observed case-mix intensity within each study group. Overall, missing cost data were limited and addressed in a manner designed to maximize the completeness of health care utilization capture while preserving internal validity and transparency of cost attribution.

All analyses were conducted using Microsoft Excel [28] and Stata version 18.5 (StataCorp LLC) [29] and following a similar analytic approach used in the previously published trial analyses [3].

## Results

### Main Findings

**Figure 1.**
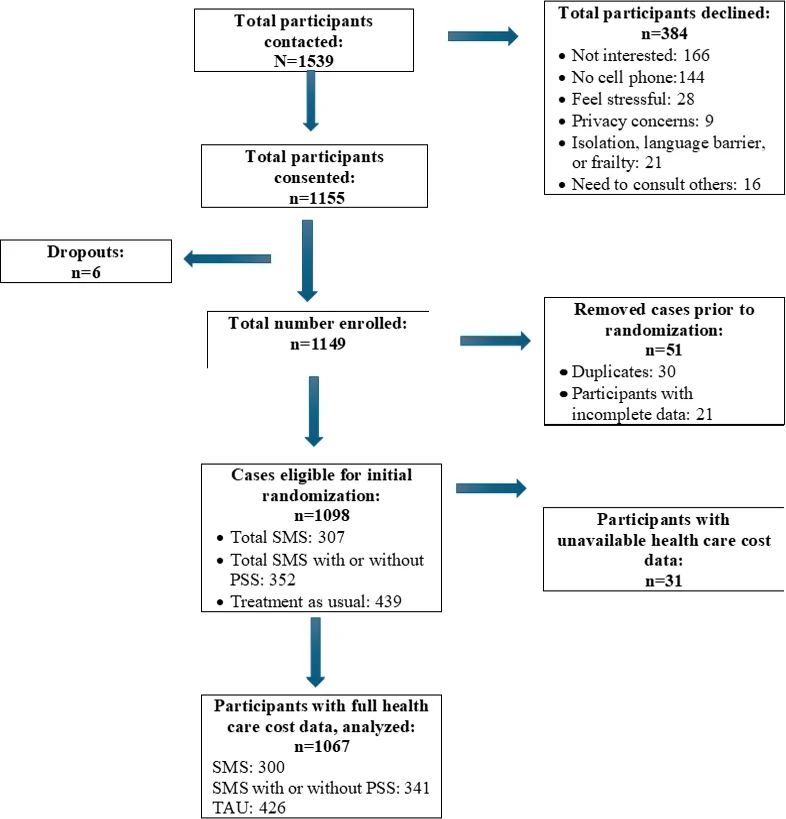
Flowchart of the study. PSS: peer support services; SMS: supportive text messaging; TAU: treatment as usual.

**Figure 2.**
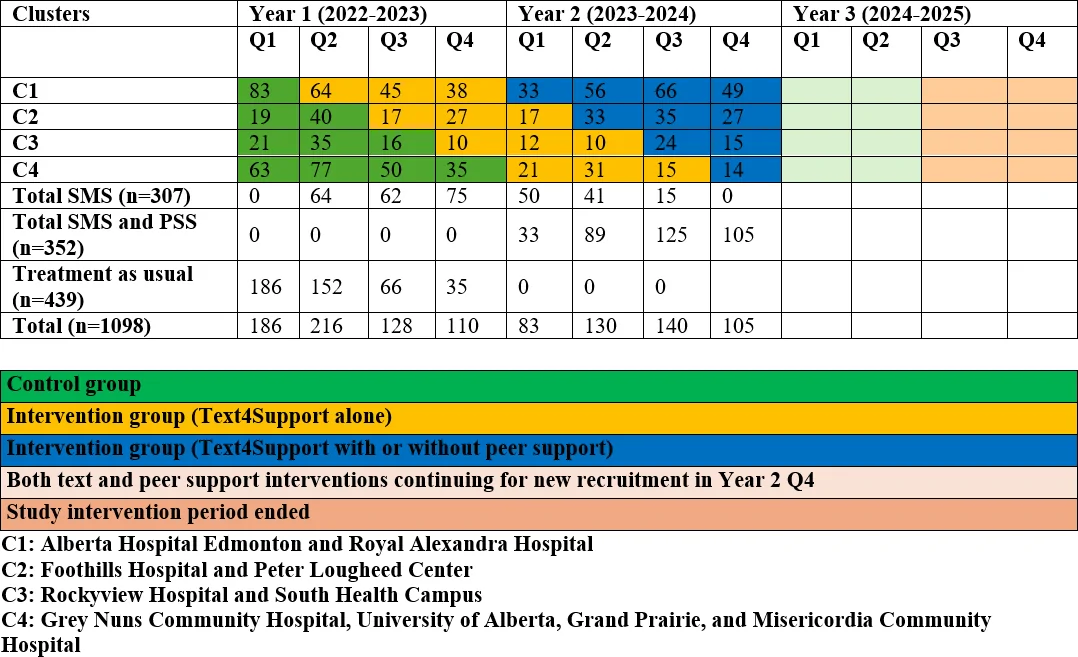
Study recruitment cluster and intervention group allocation. PSS: peer support services; SMS: supportive text messaging.

**Figure 3.**
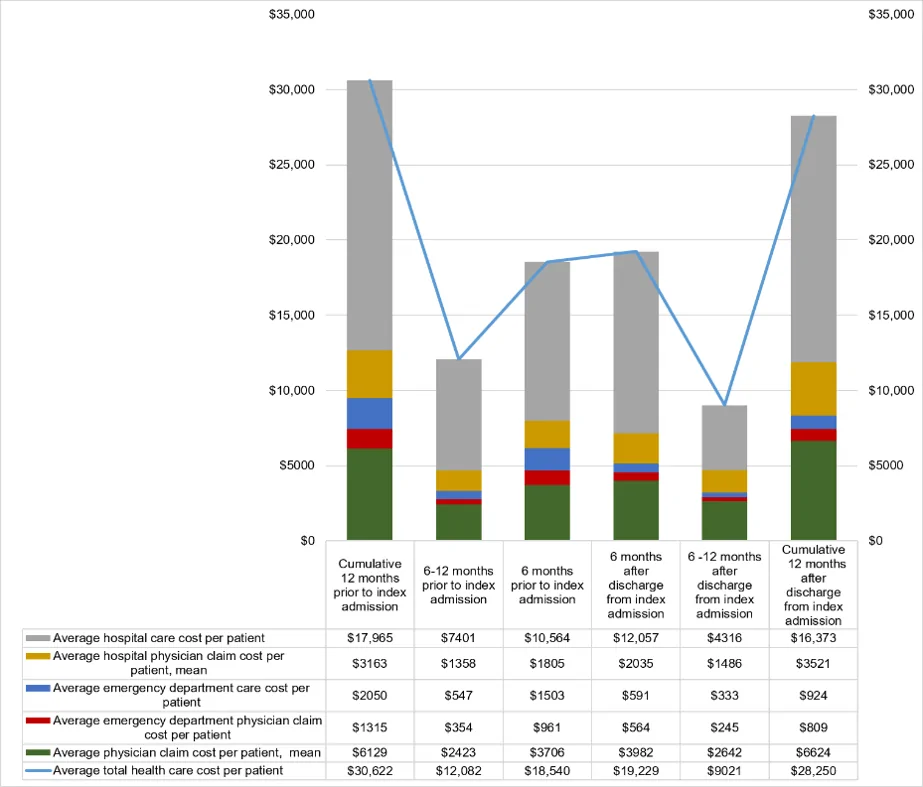
Mean health care cost (in CAD $) per patient by category, study group, and time period: treatment as usual group (TAU). ED: emergency department.

**Figure 4.**
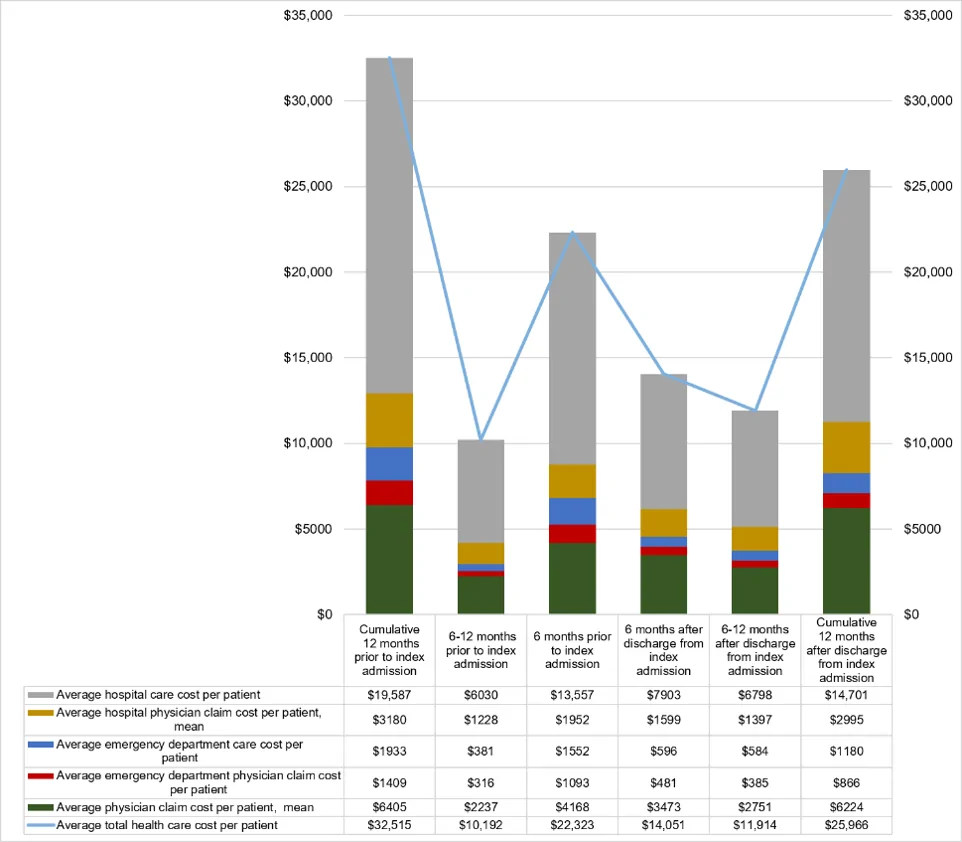
Mean health care cost (in CAD $) per patient by category, study group, and time period: supportive text messaging (SMS) group. ED: emergency department.

**Figure 5.**
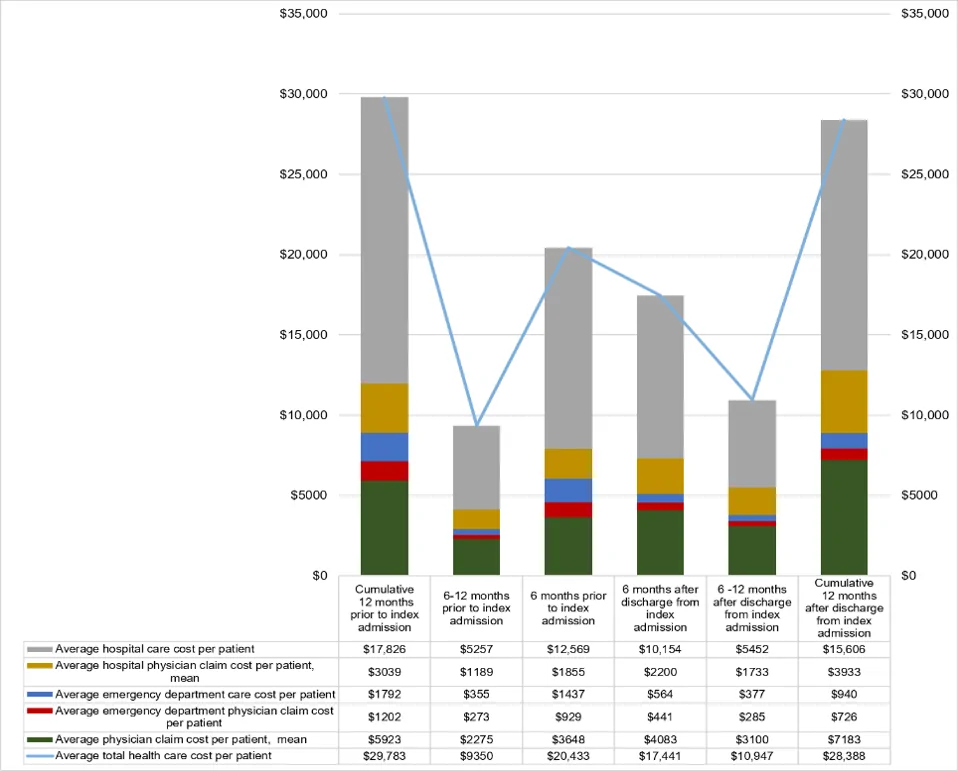
Mean health care cost (in CAD $) per patient by category, study group, and time period: supportive text messaging with or without peer support service group (SMS with or without PSS). ED: emergency department.

Baseline comparability across the 3 study groups (TAU, SMS, and SMS with or without PSS) was assessed using a one-way ANOVA. Table 1 shows that participants were comparable across groups with respect to gender, ethnicity, educational attainment, and primary presenting problem. However, a significant difference was observed for age, with participants in the TAU group being older on average than those in the SMS and SMS with or without PSS groups. As previously published in the related health care utilization paper [3], Figure 1 shows the study flowchart, while Figure 2 depicts the unit cluster randomization. Comparing average per-participant costs for each of 6 health care cost categories (hospital care cost, ED care cost, total physician cost, hospital physician cost, ED physician cost, and total health care cost), no statistically significant differences in baseline and post discharge were observed across groups for any of the cost categories (all *P* >.05; Figures 3-5 and Multimedia Appendix 1).

**Table 1.** Distribution of demographic and clinical characteristics of the participants (N=1067).

Variables	SMS^a^ (n=300), n (%)	SMS+PSS^b^ (n=341), n (%)	TAU^c^ (n=426), n (%)	*P* value
Age (y)				.001
≤25	110 (36.7)	168 (49.3)	115 (27.0)	
26-40	105 (35.0)	97 (28.4)	163 (38.3)	
>40	85 (28.3)	76 (22.3)	148 (34.7)	
Sex				.43
Male	121 (40.3)	140 (41.1)	191 (44.8)	
Female	173 (57.7)	189 (55.4)	222 (52.1)	
Other	6 (2.0)	12 (3.5)	13 (3.1)	
Ethnicity				.16
Caucasian	182 (60.7)	200 (58.7)	276 (64.8)	
Indigenous	34 (11.3)	24 (7.0)	40 (9.4)	
Black	33 (11.0)	40 (11.7)	43 (10.1)	
Asian	28 (9.3)	51 (14.9)	42 (9.9)	
Mixed/other	23 (7.6)	26 (7.6)	25 (5.9)	
Education level				.14
Less than high school	7 (2.3)	12 (3.5)	20 (4.7)	
High school diploma	156 (52.0)	188 (55.1)	208 (48.8)	
Postsecondary education	123 (41.0)	131 (38.4)	189 (44.4)	
Prefer not to say	14 (4.7)	10 (2.9)	9 (2.1)	
Primary diagnostic category				.98
Anxiety/depression	179 (59.7)	203 (59.5)	255 (59.9)	
Psychosis	46 (15.3)	58 (17.0)	69 (16.2)	
Other (including alcohol substance use and personality disorder)	75 (25.0)	80 (23.5)	102 (23.9)	

a SMS: supportive text messaging.

b PSS: peer support service.

c TAU: treatment as usual.

Subsequently, DID analyses were conducted for all 6 health care cost categories (Table 2). Initial DID screening was performed using ANOVA-based DID models to test group-by-period interactions. Statistically significant DID effects were identified for 6 months of hospital care costs (*P*=.03) and 6 months of total health care costs (*P*=.03).

For these outcomes, additional diagnostic and post hoc analyses were undertaken. We evaluated the impact of the SMS and SMS with or without PSS interventions on health care costs using a DID framework with a multiarm comparison. Because the study included more than one intervention group, DID was implemented using a 2-stage approach, which allows for the direct comparison of cost changes across all study arms, including comparisons between the 2 intervention groups. Following overall tests of group differences, post hoc pairwise comparisons of mean DID values were conducted to identify specific between-group differences, using 2-sample *t* tests with pooled variance estimates. Pairwise contrasts included SMS vs TAU (primary comparison of interest), SMS with or without PSS vs TAU, and SMS vs SMS with or without PSS. The results are reported as mean DID differences with corresponding 95% CIs and *P* values. For interpretability, contrasts are oriented so that negative values indicate cost reductions associated with the intervention. *P*<.05 was considered statistically significant (Table 3).

**Table 2.** Difference-in-difference mean health care costs 6 and 12 months preindex and postindex admission.

Group	Count	6-month difference (post-pre; CAD $), mean (SD)	12-month difference (post-pre; CAD $), mean (SD)
Hospital care cost			
TAU^a^	426	1493 (35,415)	−1592 (42,979)
SMS^b^	300	−5654 (32,088)	−4887 (41,133)
SMS with or without PSS^c^	341	−2415 (38,169)	−2221 (44,727)
*P* value		.03	.58
Emergency department care cost			
TAU	426	−912 (1546)	−1126 (2024)
SMS	300	−957 (1643)	−753 (3336)
SMS with or without PSS	340	−873 (1441)	−852 (1949)
*P* value		.79	.10
Hospital physician cost			
TAU	426	230 (5883)	346 (9836)
SMS	300	−354 (5934)	−185 (7777)
SMS with or without PSS	341	347 (6584)	894 (9659)
*P* value		.30	.34
Emergency department physician cost			
TAU	426	−397 (1651)	−506 (2042)
SMS	300	−612 (1448)	−543 (1857)
SMS with or without PSS	341	−488 (1267)	−476 (1742)
*P* value		.16	.91
Total physician cost			
TAU	426	276 (7263)	259 (11,739)
SMS	300	−692 (7298)	−181 (9828)
SMS with or without PSS	341	435 (7390)	1259 (11,508)
*P* value		.12	.24
Total health care cost			
TAU	426	857 (41,779)	−2460 (53,543)
SMS	300	−7303 (38,892)	−5821 (48,903)
SMS with or without PSS	341	−2853 (43,517)	−1813 (54,253)
*P* value		.03	.59

a TAU: treatment as usual.

b SMS: supportive text messaging.

c PSS: peer support service.

**Table 3.** Difference-in-difference estimates of mean hospital care costs and total health care costs relative to the treatment as usual (TAU) group.

Comparison group and time point	Change in cost (CAD $) (95% CI)	*P* value
Hospital care cost		
SMS^a^ vs TAU		
6 months	−7147 (−12,388 to −1906)	.01
12 months	−3294 (−9660 to 3071)	.31
SMS with or without PSS^b^ vs TAU		
6 months	−3908 (−8961 to 1145)	.13
12 months	−628 (−6765 to 5509)	.84
SMS vs SMS with or without PSS		
6 months	−3239 (−8743 to 2265)	.25
12 months	−2666 (−9351 to 4019)	.43
Total health care cost		
SMS vs TAU		
6 months	−8160 (−14,307 to −2013)	.01
12 months	−3361 (−11,128 to 4405)	.40
SMS with or without PSS vs TAU		
6 months	−3710 (−9636 to 2216)	.22
12 months	646 (−6841 to 8134)	.87
SMS vs SMS with or without PSS		
6 months	−4450 (−10,905 to 2006)	.18
12 months	−4008 (−12,164 to 4149)	.34

a SMS: supportive text messaging.

b PSS: peer support service.

At 6 months post intervention, the SMS group experienced a statistically significant reduction in hospital care costs relative to the TAU group (mean DID −$7147; 95% CI −$12,388 to −$1906; *P*=.01). No statistically significant difference was observed between SMS alone and SMS with or without PSS, indicating that the addition of peer support did not confer incremental cost savings beyond SMS alone. For total health care costs, the SMS group showed a significant reduction of $8160 at 6 months after the index admission compared with the TAU group, while no significant differential effects were observed at the 12-month period after the index admission. Similar to hospital care costs, no significant reduction was observed for the SMS with or without PSS group during both the 6-month and 12-month periods relative to the TAU group.

### Results of Sensitivity Analysis

The sensitivity analyses demonstrated that the primary findings were robust across alternative intervention costing assumptions and analytic specifications. No statistically significant between-group differences in baseline health care utilization costs were identified, supporting baseline comparability across study groups. Repeated analyses using alternative model specifications and pooled variance estimators yielded consistent results, with the observed reductions in hospital care costs and total health care utilization costs associated with the SMS intervention remaining directionally and statistically stable.

## Discussion

### Principal Findings

This exploratory secondary analysis suggests that SMS may be associated with lower health care utilization costs during the 6-month period following psychiatric discharge. From a health system perspective, the observed reductions in hospital care costs and total health care costs at 6 months post discharge among participants receiving SMS alone highlight the potential for cost avoidance during a critical and resource-intensive postdischarge period. These findings are particularly relevant for publicly funded health systems facing sustained pressure to manage rising mental health–related expenditures [1,2].

The magnitude and timing of the observed cost reductions are notable. Cost differences were concentrated in the 6-month post-discharge window, a period consistently identified as having elevated risk for adverse outcomes and high service utilization [30,31]. Evidence from systematic reviews and large cohort studies demonstrates that rehospitalizations and other adverse outcomes cluster in the first months after discharge, making this period a key target for interventions aimed at improving system efficiency [30,31]. The significant reduction in hospital care costs and total health care costs in the SMS group relative to TAU suggests that this intervention may reduce reliance on expensive inpatient services when risk and resource use are greatest. In contrast, cost differences were attenuated at 12 months post discharge, indicating that the economic impact of the SMS intervention may be strongest in the active intervention period (6 mo), when readmission risk and health care utilization are highest. This temporal pattern aligns with prior evidence showing that even modest reductions in rehospitalization rates or inpatient service use during high-risk periods can translate into meaningful system-level cost avoidance, given the high unit costs associated with psychiatric inpatient care [22,32]. From a policy perspective, these findings reinforce the importance of targeting transitional care interventions to periods where marginal gains in utilization reduction are most likely to yield economic benefits.

The addition of peer support to SMS did not result in statistically significant incremental reductions in health care utilization costs beyond those observed with text messaging alone. Although peer support interventions have demonstrated benefits for recovery-oriented and psychosocial outcomes in previous studies, evidence regarding their effects on health care utilization and costs remains mixed [33,34]. This variability may partly reflect the substantial heterogeneity in peer support models across the literature, including differences in intervention intensity, duration, delivery methods, and integration within clinical services. From a health system planning perspective, these findings highlight the importance of balancing clinical, experiential, and economic objectives when allocating limited mental health resources, as interventions that improve patient-reported outcomes may not necessarily translate into short-term reductions in high-cost service utilization.

The economic relevance of SMS is further strengthened by its low implementation cost and scalability. Digital interventions of this nature typically involve minimal marginal costs once established and can be deployed at scale without substantial additional infrastructure [7,35]. Sensitivity analyses incorporating conservative per-patient implementation costs did not alter the direction or statistical significance of the primary findings, suggesting that the observed cost avoidance is robust to plausible variations in program delivery costs. This characteristic is particularly attractive in resource-constrained health systems seeking cost-effective strategies to enhance postdischarge care.

From a policy and service delivery perspective, these findings suggest that integrating SMS into routine discharge planning may represent a pragmatic approach to improving allocative efficiency in mental health care, especially in health systems where transitions from inpatient to outpatient care do not consistently include timely access to follow-up services or continuity of care. Even relatively small per-patient reductions in hospital-based costs, when applied across large populations of discharged psychiatric patients, may yield substantial system-level economic impacts [1,2].

Several considerations and potential limitations should be noted when interpreting these results. First, although the original protocol proposed a cost-utility analysis using quality-adjusted life years derived from EQ-5D-5L data, this analysis could not be undertaken because of low follow-up completion rates for the EQ-5D-5L surveys, limiting the availability of reliable health use data for longitudinal analysis. Second, while SMS was associated with lower health care utilization costs during the active intervention period, the present analysis did not incorporate full intervention delivery costs into the primary economic model, and therefore, conclusions regarding net cost savings should be interpreted cautiously. Third, the observed economic effects were concentrated within the 6-month post-discharge period during which patients received the active intervention. This pattern suggests that SMS may be most effective as a time-limited, transitional intervention, rather than a stand-alone strategy conferring sustained economic benefits beyond the period of active support. Future research should examine whether extended intervention duration or integration with additional postdischarge supports could enhance longer-term economic impact. Fourth, although the analyses focused primarily on hospital care costs and total health care costs as the most policy-relevant outcomes, formal correction for multiple comparisons was not applied; therefore, findings relating to secondary cost categories should be interpreted cautiously and considered exploratory. Fifth, this analysis was conducted from a health system perspective and did not capture broader societal costs, such as patient out-of-pocket health care expenditures, caregiver burden, or productivity losses. Incorporating these dimensions in future economic evaluations may further clarify the full-value proposition of digital postdischarge interventions. Sixth, although baseline health care utilization costs were comparable across study groups, the available administrative data did not permit formal multiperiod assessment of preintervention trends; therefore, the parallel trends assumption underlying the DID analyses could not be fully verified.

Overall, this study contributes policy-relevant evidence that a low-cost, scalable digital intervention can reduce high-cost health care utilization during a critical postdischarge period, following psychiatric hospitalization. By demonstrating measurable reductions in the hospital and total health care costs without substantial additional investment, these findings support the consideration of SMS as a component of value-based, recovery-oriented mental health care delivery [3,9].

### Implications for Policy, Practice, and Future Research

#### Implications for Policy and Practice

The findings of this study have important implications for mental health policy and service delivery within publicly funded health systems. The demonstrated short-term cost reductions associated with SMS highlight its potential role as a cost-efficient transitional care intervention following psychiatric discharge. Given that the 6-month postdischarge period represents a phase of heightened vulnerability, resource use, and readmission risk, policies that prioritize low-cost, scalable supports during this window may yield meaningful improvements in allocative efficiency without compromising care quality.

From a policy perspective, the results support the integration of automated SMS into routine discharge planning as a standard adjunct to usual care. Because the intervention is inexpensive to implement, requires minimal infrastructure, and can be delivered at scale, it is well aligned with value-based health system objectives that emphasize maximizing population impact per dollar invested. However, an important finding of this study was that the observed reductions in health care utilization costs were confined to the 6-month period during which participants actively received SMS and were no longer evident at 12 months. This pattern suggests that the economic benefits of the intervention may diminish after active support ends, highlighting the potential need for longer-duration interventions or ongoing engagement to sustain reductions in health care utilization. Importantly, the absence of incremental cost benefits associated with adding peer support suggests that, where resources are constrained, SMS alone may represent the most economically efficient option for broad postdischarge implementation. Health authorities may therefore consider reserving more resource-intensive PSSs for targeted subgroups based on clinical needs or recovery goals, rather than as a universal add-on.

#### Implications for Future Research

Future research should aim to refine and extend these findings in several ways. First, longer-term evaluations are needed to determine whether periodic “booster” messaging or extended-duration digital support can sustain economic benefits beyond the initial postdischarge period. Second, subgroup analyses could help identify patient populations most likely to benefit economically from SMS, enabling more targeted deployment. Third, future economic evaluations should incorporate broader societal perspectives, including productivity losses, caregiver burden, and patient out-of-pocket costs, to capture the full value of digital transitional interventions. Finally, comparative studies examining the cost-effectiveness of SMS relative to other transitional care models would further inform optimal investment decisions in mental health systems.

### Conclusion

This secondary exploratory study suggests that SMS may represent a promising strategy for reducing health care utilization costs, following psychiatric discharge. By demonstrating that these economic benefits may be concentrated within the first 6 months post discharge (when readmission risk and service utilization are greatest), the findings highlight the potential value of targeted, time-limited digital supports as part of transitional mental health care. The absence of significant cost differences at 12 months suggests that the economic benefits of SMS may be largely confined to the active intervention period. Future randomized trials evaluating longer-duration interventions, including 12-month SMS programs with prospectively planned economic evaluations, are warranted to determine whether sustained delivery can produce longer-term reductions in health care utilization and associated costs. The absence of demonstrable additional cost savings from combining text messaging with peer support underscores the importance of aligning intervention intensity with system-level economic objectives. Overall, these results support considerations for the integration of automated SMS into routine discharge pathways as a pragmatic, value-based strategy to reduce the economic burden of psychiatric inpatient care in publicly funded health systems. Notwithstanding, the findings should be interpreted within the context of the study population, which consisted of adults discharged from acute psychiatric inpatient care across urban psychiatric service settings in Alberta, Canada. Participants represented a clinically complex population at elevated risk of readmission and recurrent health care utilization, suggesting that the observed reductions in health care utilization costs associated with SMS may be particularly relevant to high-risk transitional psychiatric care populations within publicly funded health systems. This study should also be interpreted as a secondary exploratory economic analysis. Future prospective economic evaluations incorporating intervention delivery costs, health utility outcomes, and prespecified statistical approaches will be important to validate these observations.

## Supplementary material

10.2196/93954 Multimedia Appendix 1 Average cost by category per participant across all 3 study groups.

## Abbreviations

DID: difference-in-difference
ED: emergency department
PSS: peer support service
RIW: resource intensity weight
SMS: supportive text messaging
TAU: treatment as usual
