# Supplementary material for: Association of Supportive Text Messaging With Health Service Utilization Costs Following Psychiatric Admission: Secondary Economic Analysis of a Stepped-Wedge Cluster Randomized Trial
Source: JMIR Ment Health. 2026 Aug 21;13:e93954. doi: 10.2196/93954 (PMC13495993; doi:10.2196/93954)
Supplement: Supplementary file 1 [file mental-v13-e93954-s001.docx]

**Appendix 1:** Average cost by category per participant across all three study groups, CAD

| **Groups** | **Count** | **Prio index admission** | | **Post discharge after index admission** | |
| --- | --- | --- | --- | --- | --- |
|  |  | **12-months** | **6-months** | **6-months** | **12-months** |
| **Hospital Care Cost** | | | | | |
| TAU | 426 | 17,965 | 10,564 | 12,057 | 16,373 |
| SMS | 300 | 19,587 | 13,557 | 7,903 | 14,701 |
| SMS+PSW | 341 | 17,826 | 12,569 | 10,154 | 15,606 |
| ***p-value*** |  | *0.728* | *0.142* | *0.232* | *0.849* |
| **Emergency Department Care Cost** | | | | | |
| TAU | 426 | 2,050 | 1,503 | 591 | 924 |
| SMS | 300 | 1,933 | 1,552 | 596 | 1,180 |
| SMS+PSW | 341 | 1,792 | 1,437 | 564 | 940 |
| ***p-value*** |  | *0.235* | *0.524* | *0.960* | *0.426* |
| **Total Physician Cost** | | | | | |
| TAU | 426 | 6,365 | 3,706 | 3,982 | 6,624 |
| SMS | 300 | 6,405 | 4,168 | 3,476 | 6,224 |
| SMS+PSW | 341 | 5923 | 3,648 | 4,083 | 7,183 |
| ***p-value*** |  | *0.738* | *0.438* | *0.441* | *0.512* |
| **Hospital Physician Cost** | | | | | |
| TAU | 426 | 3,185 | 1,805 | 2,035 | 3,521 |
| SMS | 300 | 3,180 | 1,952 | 1,599 | 2,995 |
| SMS+PSW | 341 | 3,039 | 1,855 | 2,200 | 3,933 |
| ***p-value*** |  | *0.944* | *0.902* | *0.304* | *0.361* |
| **Emergency Department Physician Cost** | | | | | |
| TAU | 426 | 1,315 | 961 | 564 | 809 |
| SMS | 300 | 1,409 | 1,093 | 481 | 866 |
| SMS+PSW | 341 | 1,202 | 929 | 441 | 726 |
| ***p-value*** |  | *0.308* | *0.205* | *0.409* | *0.639* |
| **Total Healthcare Cost** | | | | | |
| TAU | 426 | 26,380 | 15,773 | 16,630 | 23,921 |
| SMS | 300 | 27,925 | 19,277 | 11,974 | 22,105 |
| SMS+PSW | 341 | 25,542 | 17,654 | 14,800 | 23,729 |
| ***p-value*** |  | *0.751* | *0.203* | *0.265* | *0.870* |
